# Gunshot Wounds

**Published:** 1900-06-02

**Authors:** 


### GUNSHOT WOUNDS.

Speaking at the Royal Medical and Chirurgical Society, Sir William MacCormac related many instances of the extraordinary gunshot injuries which had been recovered from in South Africa, and pointed out some of the lessons taught as to the treatment of such wounds.

### The Character of the Wounds.

In general character the Lee-Metford and Mauser wounds, as compared with those he had seen before, were extraordinarily mild. The great infrequency with which articles of clothing or any foreign material was carried in was also noteworthy, for this used to be a very common occurrence in former times. Lodgement of the bullets was met with in a much larger proportion of the cases than was to have been expected, considering the velocity at which they often travelled.

### Abdominal Injuries.

Many enthusiastic young surgeons, officers in the Royal Army Medical Corps and others, went out full of hope of performing successful abdominal operations, but recent experience had served to confirm the opinion he had formed from previous experience of the conditions met with in actual warfare, that the prospects of success in abdominal surgery were anything but bright. He well remembered that surgical genius Marion Sims being full of the same hope on the eve of the battles of 1870. They had determined—and the previous teaching largely justified the conclusion—to operate in those cases where the abdomen was without doubt traversed by a bullet, and where the sufferer seemed in a fit condition to be operated upon. This opinion, however, must now be changed. He had now seen more than 50 cases of injury of this kind recover without any interference beyond antiseptic occlusion, and in many instances not even that was accomplished; while comparatively few cases had recovered after operation in this war, and none during the war in Cuba. A

very useful hint as to the advantage of an empty stomach, both before and after wound of the abdomen, was given by a case which he related of an officer of the Royal Army Medical Corps who was wounded while dressing a wounded man near the front of the fighting line. He was able to discover almost immediately where the bullet had entered in front—through the rectus muscle, about one inch below the right costal margin and one and a half inches from the median line—but he could not at the time ascertain where it had emerged behind, which was over the right sacro-iliac synchondrosis. He had had nothing to eat or drink for ten hours previously, and then only a little porridge; and, although he believed he was mortally wounded, he thought his best chance was to remain absolutely still, and this he did all the afternoon and far into the night, until the stretcher-bearers arrived 12 long weary hours afterwards. All this time he was watching and waiting for the certain advent of symptoms of peritonitis. None, to his surprise, came, but the distress, mainly in the form of pain in the back, from the constrained position was terribly hard to bear. During the night some passing Boers offered him water to drink; yet, consumed though he was with a raging thirst, he declined it, and begged two similarly wounded soldiers lying near him, also shot through the abdomen, to refuse likewise. They did not, however, show the same resolution; they drank copiously, and one died beside Captain —— during the night after great agony, and the other the day afterwards in hospital. "Words fail me," said Sir William, "to describe adequately the horror of such a situation or the courage and heroic endurance of such a man. He made a complete recovery, as truly he deserved to do."

### Wounds of the Chest.

In the case of chest wounds, the principle of non-interference and antiseptic occlusion were most strongly affirmed. A large proportion of these cases recovered often without noteworthy symptoms. There were many astonishing instances of recovery after shot had passed through one, and in some instances both lungs. The same thing was observed during the Cuban War. Some very curious cases were observed in which, although it seemed impossible to understand how the heart had escaped unless the bullet were deflected, a good recovery, nevertheless, ensued. There was nothing in any of these cases to indicate that the bullets had not traversed in a perfectly straight line from the point of entrance to the point of exit.

### The Question of Removal of Bullets.

Sir William MacCormac spoke strongly in favour of letting well alone. It was, he said, a surprising thing that so many bullets lodged considering the immense velocity with which they travelled. The question was, what ought to be done in such cases? If the bullet were causing irritation, producing pain, or were likely from its position to be a source of future disturbance, by all means let it be excised if this could be done without undue risk; but in many cases it caused no inconvenience whatever, and in these cases he was sure that it should be left alone, unless, perhaps, when lying immediately under the skin. He mentioned two cases in which death from hæmorrhage followed ineffective and protracted attempts to remove projectiles; and another in which the search had to be abandoned after the pleural sac had narrowly escaped being opened. In this case he asked why the operation had been done, and was told that "the man wished the bullet taken out." No other reason was given. Sir William said that he referred to this matter because he saw that this hunting for bullets was once more being recommended and that a considerable proportion of the operations done at certain of the hospitals were those for extraction of bullets or their fragments.

